# Smell disorders associated with COVID-19 infection

**DOI:** 10.1186/s43163-021-00095-9

**Published:** 2021-05-01

**Authors:** Mohammad Waheed El-Anwar, Sherif Mamdoh Mohamed, Ahmed Hassan Sweed

**Affiliations:** Otorhinolaryngology, Head and Neck Surgery Department, Faculty of Medicine, Zagazig University, Zagazig, Egypt

**Keywords:** COVID-19, Coronavirus, Anosmia, Hyposmia, Smell, Olfactory, ENT, Otorhinolaryngology

## Abstract

**Background:**

We performed a search in the PubMed databases, Web of Science, LILACS, MEDLINE, SciELO, and Cochrane Library using the keywords COVID-19, Novel coronavirus, corona, 2019-nCoV, SARS-CoV-2, ENT, nose, anosmia, hyposmia, smell, olfactory, ORL, different ENT related symptoms. We reviewed published and peer-reviewed studies that reported the ENT manifestations in COVID-19 laboratory-confirmed positive patients.

**Main text:**

Within the included 2549 COVID-19 laboratory-confirmed positive patients, smell affection was reported in 1453 patients (57%). The other reported ENT manifestations were taste disorder (49.2%), headache (42.8%), nasal blockage (26.3%), sore throat (25.7%), runny nose or rhinorrhea (21.3%), upper respiratory tract infection (URTI) (7.9%), and frequent sneezing (3.6%).

**Conclusion:**

Smell affection in COVID-19 is common and could be one of the red flag signs in COVID-19 infection. With a sensitivity of utilized questionnaire in smell identification, a homogenous universal well-defined COVID-19 questionnaire is needed to make the COVID-19 data collection more sensible.

## Background

At December 2019, the severe acute respiratory syndrome coronavirus 2 (SARS-CoV-2), initially known as the 2019 novel coronavirus (2019-nCoV), started in China in Wuhan [1, 2]. Since that moment, this novel virus, also named as coronavirus disease 2019 (COVID-19), has crossed all countries’ borders with dramatic spread all over the world till the World Health Organization (WHO) defined it as a pandemic disease on March 11, 2020.

The COVID-19 is part of the species of the SARS-related coronaviruses that over the last two decades have led to preceding epidemics as SARS-CoV in China in 2002–2003 [3] and the Middle East Respiratory Syndrome (MERS-CoV) in Saudi Arabia in 2012–2013 [4].

The novel COVID-19 is presented mainly by lower respiratory tract-related manifestations such as fever, cough, dyspnea, and chest tightness that could progress quickly to acute respiratory distress syndrome (ARDS) [5]. However, COVID-19 leads as well to different upper respiratory tract-related manifestations comprising sore throat, smell dysfunction, and nasal congestion [6].

The available data on the ear nose throat (ENT) manifestations of COVID-19 is recently published [7] and review studies on smell in COVID-19-positive patients are still growing.

Smell and taste dysfunctions in COVID-19 patients were sparsely cited in the literature and there is still a paucity of peer-reviewed publications to support a causal association between COVID-19 and anosmia [7, 8].

Thus, the aim of the current work was to detect and discuss the smell disorders associated with confirmed COVID-19 infection in the reviewed and published literature.

## Main text

### Methods

We searched several medical databases, including PubMed databases, Web of Science, LILACS, MEDLINE, SciELO, and Cochrane Library at July 2020 to find out relevant articles. We used the following keywords: COVID19, COVID-19, Novel Coronavirus, corona, 2019-nCoV, SARS-CoV-2, anosmia, hyposmia, smell, olfactory, smell dysfunction, nose, and different ENT related symptoms.

We focused our review on studies reporting the smell disorders in laboratory-confirmed positive COVID-19 patients. We included studies that asked about and demonstrated the incidence smell disorders among other COVID-19 manifestations such as fever, cough, and shortness of breathing in laboratory-confirmed positive COVID-19 patients. Non-published studies, studies that were not published in indexed journals or published without peer review, studies that included suspected cases beside confirmed cases, studies that did not ask about or test for smell disorders, and studies that are not available in English language were excluded from the current review. Then the authors collected, tabulated, and analyzed the results of the studies that met these inclusion and exclusion criteria.

## Results

Between a large number of read COVID-19 papers, 23 reviewed and published studies met the inclusion and exclusion criteria of the present review and reported smell disorders in COVID-19-positive patients [9–31] (Table 1). These studies included 2549 laboratory-confirmed positive COVID-19 patients, 1107 females (43.4%) and 838 males (32.8%). While in 603 included patients, the gender was not mentioned and one patient was gender diverse. Smell disorder was detected in 1453 patients (57%). In 648 patients of them, type of smell disorders was defined as anosmia in 376 patients (58%), hyposmia in 270 patients (41.6%), dysosmia in 2 patients (0.4%).

**Table 1:** All included studies discussing olfactory dysfunction with COVID-19

	Article	Publish date	Type or article	COVID-19 (M/F)	COVID-positive patient with smell disorder				Percentage	Smell identification	Notes
					Smell disorder	Anosmia	Hyposmia	Dysosmia			
1	Giacomelli et al [9]	Mar 2020	Cross-sectional study	59(40/19)	17	Unknown			28.8%	Questionnaire	
2	Vaira et al [10]	Apr 2020	Rapid communication paper	320	62	Unknown			19.4%	Questionnaire	
3	Villalba et al [11]	Apr 2020	Case series	2(1/1)	2	Unknown			100%	Questionnaire	
4	Hjelmesæth et al [12]	Apr 2020	Case series	3 (2/1)	3	Unknown			100%	Questionnaire	
5	Lechien et al [13]	Apr 2020	Cross-sectional multicenter study	417 (154/263)	357	284	73		85.6%	Questionnaire	
6	Mao et al [14]	Apr 2020	Retrospective study	214 (87/127)	11	Unknown			5.1%	Questionnaire	
7	Gilani et al [15]	Apr 2020	Case series	5 (2/3)	5	Unknown			100%	Questionnaire	
8	Eliezer et al [16]	Apr 2020	Case report	1 (0/1)	1	Unknown			100%	CCCRC	CT scan of the nasal cavity showed bilateral inflammatory obstruction of the olfactory clefts that was confirmed on MRI of the nasal cavity. There were no anomalies of the olfactory bulbs and tracts.
9	Yan et al [17]	Apr 2020	Cross-sectional study	59 (29/29/1)	40	Unknown			67.8%	Questionnaire	Comparison between probability of acute smell loss in COVID-19 and normal individual
10	Klopfenstein et al [18]	Apr 2020	Retrospective study	144	54	Unknown			37.5%	Questionnaire	
11	Moein et al [19]	Apr 2020	Case-control study	60 (20/40)	59	15	44		98.3%	UPSIT^a^	Smell disorder identification by history 21 patients / by test 59 patients
12	Spinato et al [20]	Apr 2020	Cross-sectional study	202 (97/105)	130	48	82		64.4%	Questionnaire	
13	Beltrán-Corbellini et al [21]	Apr 2020	Case-control study	79	25	14	9	2	31.6%	Questionnaire	Comparison between probability of acute smell loss in COVID-19 and influenza patient
14	Yan et al [22]	Apr 2020	Retrospective study	128 (61/67)	75	Unknown			58.6%	Questionnaire	
15	Kaye et al [23]	Apr 2020	Short communication study	237 (108/129)	237	Unknown			100%	Questionnaire	
16	Ottaviano et al [24]	Apr 2020	Case series	6	6	Unknown			100%	Le Nez du Vin^c^	
17	Heidari et al [25]	Apr 2020	Case series	23 (8/15)	23	Unknown			100%	Questionnaire	
18	Kim et al [26]	May 2020	Cross-sectional study	172 (66/106)	68	Unknown			39.5%	Questionnaire	
19	Boscolo-Rizzo et al [27]	May 2020	Cross-sectional study	54	34	Unknown			63%	Questionnaire	Comparison between probability of acute smell loss in COVID-19 and normal individual
20	Luers et al [28]	May 2020	Retrospective study	72 (41/31)	53	Unknown			73.6%	Questionnaire	
21	Vaira et al [29]	May 2020	Cross-sectional study	33 (11/22)	17	13	4		51.5%	CCCRC^b^	
22	Vaira et al [30]	Jun 2020	Cross-sectional study	72 (27/45)	61	2	58		84.7%	CCCRC^b^	^b^Smell disorder identification by history 44 patients / by test 61 patients
23	Boscolo-Rizzo et al [31]	Jul 2020	Cross-sectional study	187 (84/103)	113	Unknown			60.4%	Questionnaire	
				2549 M = 838 F = 1107 Unknown = 603 Gender diverse = 1	1453	376	270	2	57%		

^a^University of Pennsylvania Smell Identification Test (UPSIT)

^b^Connecticut Chemosensory Clinical Research Center (CCCRC) orthonasal olfaction test'

^c^Le Nez du Vin = six supra-threshold odors forced multiple choice smell identification test

**Fig. 1:**
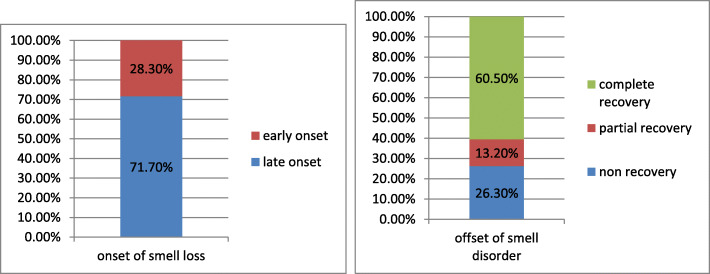
Onset/offset of smell disorder with COVID-19 patients; onset (early-late)–offset (complete recovery-partial recovery-persistent smell disorder)

Smell affection was early (first presentation/only presentation) in 337 patients (28.3%) and was late (3 to 5 days after the initial symptoms of the acute respiratory tract infection) in 807 patients (71.7%) (Fig. 1). Only 0.9% of cases with early smell disorder suffered from severe to critical COVID-19 sequelae; on the other hand, 4.23% of cases with late smell dysfunction got the worst form of COVID-19 disease.

Early (complete) recovery of smell (within 2 weeks) was reported in 556 patients (60.5%), while late (partial) recovery was detected in 121 patients (13.2%). On the other hand, in 242 patients (26.3%), smell did not recover (Fig. 1).

Olfactory evaluation was performed by questionnaire in most patients (1281 patients, 88.2%), while smell test was used in 172 patients (11.8%). The utilized smell identification tests were University Of Pennsylvania Smell Identification Test (UPSIT), Connecticut Chemosensory Clinical Research Center orthonasal olfaction test (CCCRC) and Le Nez du Vin “six supra-threshold odors forced multiple choice smell identification test.” There is discrepancy in smell identification results between history-questionnaire evaluation and other tests. Among those 172 COVID-19 patients evaluated by smell identification tests, 160 patients proved to have smell disorder; either anosmia or variable degree of hyposmia according to the scale in the aforementioned smell identification tests, and only 105 patients had smell disorder as main complaint. So, sensitivity of questionnaire in smell identification (105/160 = 65.6%).

When we reviewed the smell affection in relation to the severity of COVID-19 disease, we found that 921 smell-affected patients (96.23%) were asymptomatic or had mild to moderate COVID-19 condition “No hospitalization-No oxygen therapy,” 29 smell-affected patients (3.02%) had severe COVID-19 disease, and 7 smell-affected patients were critical COVID-19 (0.73%).

**Fig. 2:**
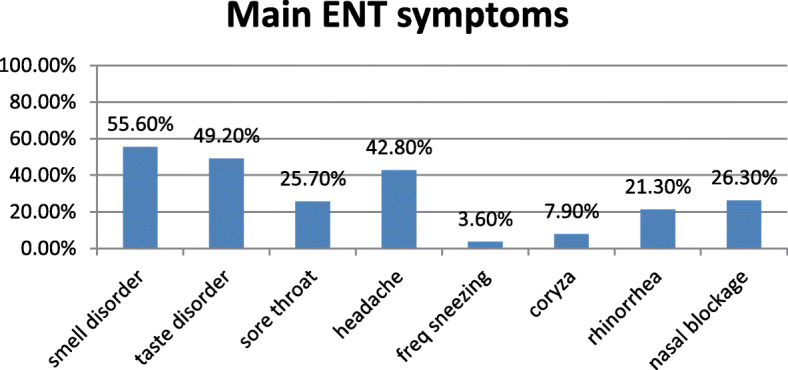
ENT symptomatology of COVID-19

**Fig. 3:**
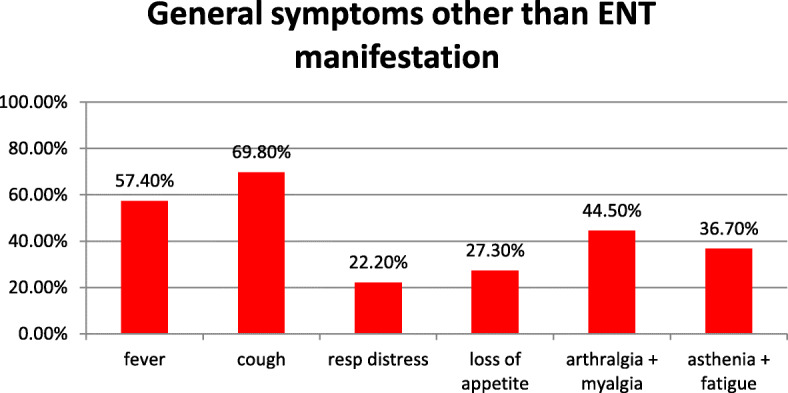
General manifestations other than ENT symptoms in COVID-19

In 1515 included patients, complete ENT symptomatology was recorded in which smell disorder was detected in 843 patients (55.6%), and taste disorder was detected in 746 patients (49.2%). Meanwhile, other ENT manifestations were runny nose or rhinorrhea reported in 323 patients (21.3%), nasal congestion and blockage manifested in 398 patients (26.3%), sore throat reported in 420 patients (25.7%), headache presented in 648 patients (42.8%), sneezing in 54 patients (3.6%), and URTI reported in 119 patients (7.9%) (Fig. 2), while non-ENT manifestations in those patients were fever in 869 patients (57.4%), cough in 1058 patients (69.8%), dyspnea in 336 patients (22.2%), arthralgia/myalgia in 674 patients (44.5%), asthenia/fatigue in 556 patients (36.7%), and loss of appetite in 414 (27.3%) (Fig. 3).

## Discussion

A novel coronavirus (CoV) epidemic, produced by the severe acute respiratory syndrome coronavirus-2 (SARS-CoV-2), began in December 2019 from China [4, 7]. On February 11, 2020, the WHO named the disease caused by this novel virus as COVID-19. The widespread transmission and infectivity of COVID-19 marks it as a significant pathogen with a considerable health threat [32].

COVID-19 manifests with a wide clinical range extending from no symptoms to multi-organ dysfunctions and septic shock. Despite its rapid distribution worldwide, the clinical features of COVID-19 remain to a variable extent non-specific [6–8, 32].

The nasal, nasopharyngeal, and/or the orpharyngeal tissues are part of the main harbor locations of the infection, main site of sample taking for testing, and a main source of infection transmission. However, most COVID-19 published studies are focused on the lower respiratory tract manifestation and sequels because of their life-threatening potentiality [7, 32].

Meanwhile, the literature on ENT manifestation during COVID-19 infection remains growing. Thus, there is value in studying smell dysfunction and other ENT manifestations of such novel virus and there is a need to recognize the defining smell dysfunction epidemiological and clinical characteristics in COVID-19 with more precision.

In the current review of literature, we tried to collect the data concerning the smell disorders associated with laboratory-confirmed COVID-19 cases in only the peer-reviewed and published papers to provide an up-to-date delineation of the olfactory clinical characteristics and incidence in COVID-19 patients to help ENT and other physicians dealing with COVID-19 to understand and approach such cases and assist in data building up for this novel disease.

The results of the current study agree with previous reports [6–8] that fever (recounted in 57.4% of the included patients) and cough (reported in 69.8%) are the principal symptoms of COVID-19 whereas gastrointestinal and ENT symptoms were less common, suggesting the difference in viral tropism as compared with influenza, SARS-CoV, and MERS-CoV [4, 21] .

In the current study, the percentage of COVID-19 patients complaining of smell disorder was about 57% with the fact that questionnaire as a tool in smell identification with poor sensitivity value 65.6%. The most common ENT manifestation of COVID-19 in descending order were smell dysfunction (55.6%), taste disorder (49.2%), headache (42.8%), nasal congestion and blockage (26.3%), and sore throat (25.7%). On the other hand, the most prevalent associated general symptoms in relation to smell and taste disorder in descending order were cough (69.8%), fever (57.4%), arthralgia/myalgia (44.5%), asthenia/fatigue (36.7%), loss of appetite (27.3%), and dyspnea (22.2%).

Degree of smell affection shows variability, anosmia accounts to about 58%, while hyposmia about 41.6%.

About 28.3% of affected patients complained of early smell affection as the only or first presentation with only 0.9% possibility of progression to severe or critical COVID-19. Late smell affection occurred 3–5 days after common acute respiratory tract infection in about 71.7%.

Even though smell recovered in most cases (73.7%) whether early recovery (within 2 weeks, 60.5%) or later recovery (13.2%), smell did not recover in more than one quarter of the patients (26.3%). No specific regimen was associated with olfaction recovery. Vitamin, omega-3, and trace elements with nasal steroid were prescribed without evidence of superiority of certain medication. Olfaction recovery was reported even without medication. This must be discussed with the patients and the physicians and also there is a need to evaluate the used therapy in those patients and its effect on the smell recovery. Also the viral strain as director of the prognosis is another question.

As an overall view regarding the severity of COVID-19 and smell disorder, in less than 5% of the smell-affected patients, the COVID-19 was severe or critical. This may reflect no association between disease severity and smell affection, but smell disorder could be masked by both respiratory and critical manifestations in those severe and critical COVID-19 patients.

Post-viral anosmia is a widespread cause of adult smell dysfunction (40% of anosmia cases). Viruses that induce the common cold or upper respiratory tract infections are well known to lead to post-infectious smell loss. The previously defined corona viruses are assumed to account for 10–15% cases [33]. This novel viral infection seems to affect smell sensation over other viruses. Odds ratio describing likelihood of COVID-19 associating with smell disorder is over that of influenza virus by 4.5 folds [21]. Moreover, acute smell dysfunction is reported in COVID-19 infection over normal persons by 13.2 times (odds ratio of smell disorder in normal and COVID-19-positive persons in two studies) with exclusion of obstructive olfactory disorder [17, 27].

Smell and taste dysfunctions were sparsely declared in the COVID-19 literature, and there is still a lack of peer-reviewed literature to support a causal association between COVID-19 and anosmia [6–8]. Olfactory neuritis is the most accepted theory of smell disorder but some studies emphasize on the presence of inflammatory change in olfactory cleft to be more than the changes affecting the olfactory neural pathway which is clearly detected by radiological studies like CT-MRI [16].

In peer-reviewed studies apart from case reports, smell dysfunction showed wide variation, with high records of 98.3% [19] and 85.6% [13] and low percentage of 5.1% [14] and 19.4% [10] respectively. However, they utilized a questionnaire focused on the psychological and social burden of the smell disorders, particularly with the COVID-19 pandemic scenario and the following social life restrictions, which might result in overestimation [13].

In the current review, smell affection was detected in 57% of included patients. Most COVID-19 studies particularly at the primary spread of the disease from December 2019 to March 2020 did not mention the smell affection particularly the early and primary reports, and most COVID-19 patients (66%) reported a complete recovery of their chemosensitive functions during the disease course [23].

Zhang et al [34] recommend considering patients who complained of anosmia without runny nose or nasal obstruction COVID-19 suspicion and recommending starting testing or self-isolation for those patients.

### Limitations

Because of the rapid serious health emergency of COVID-19, data collection and analysis are very difficult due to incomplete documentation lacking universal accurate description of the clinical manifestations without using a universal questionnaire for those patients. In addition, most COVID-19 studies missed asymptomatic or mild confirmed cases managed at home and there is very limited available ENT endoscopic or radiological data in COVID-19 published papers. All these mentioned limitations are features of the published researches on COVID-19 up till now and should be taken into consideration in future research. Moreover, most smell affection was evaluated via a questionnaire and patients were identified by the reported questionnaire submitted by themselves, which were not verified by the researchers. Besides, some essential information, such as gender and age, did not appear in some studies and previous history of smell disorders and nasal diseases and/or nasal examination was not mostly mentioned. Many early COVID-19 studies did not ask about or test for smell disorders, and early questionnaire and/or checklist did not include smell disorders. Therefore, all included studies in the current review were published after March 2020. Chinese studies showed less smell mention that may be attributed to less doctors, patients, and community orientation about the condition or presence of different viral strains

Thus, we agree with El-Anwar et al [7] that a standard worldwide questionnaire for definite COVID-19 manifestations is required to make the COVID-19 data well defined, homogenous, and complete in order to deliver insights of diagnostic features of the common COVID-19 manifestations.

Thus, otolaryngologists should be mindful of the symptom of anosmia in outpatients so as not to delay the diagnosis of COVID-19.

It is highly recommended to re-assess the recovered COVID-19 patients who become negative for late disease sequels including the smell examination and nose radiology since the late sequels of the COVID-19 infection after being negative need also to be assessed. Pathogenesis is not well understood, study of olfactory bulb size in those patients may point to the pathogenesis.

Otolaryngologists, physicians, patients, and the community should be aware of anosmia to avoid delaying the diagnosis of COVID-19 and thus contributing to an epidemic.

## Conclusions

Olfactory manifestations for COVID-19 are common and should be added to suspected clinical criteria of COVID-19 particularly if nasal examination was non-significant. However, a standard universal questionnaire by well-defined COVID-19 manifestations is needed to make the COVID-19 data accurately defined, homogenous, and complete.

## Data Availability

All data generated or analysed during this study are included in this published article and its supplementary information files.

## Abbreviations

SARS-CoV-2: Severe acute respiratory syndrome coronavirus 2
2019-nCoV: 2019 novel coronavirus
COVID-19: Coronavirus disease 2019
ARDS: Acute respiratory distress syndrome
UPSIT: University Of Pennsylvania Smell Identification Test
CCCRC: Connecticut Chemosensory Clinical Research Center orthonasal olfaction test
